# Supplementary material for: OxyS small RNA induces cell cycle arrest to allow DNA damage repair
Source: EMBO J. 2017 Dec 13;37(3):413–26. doi: 10.15252/embj.201797651 (PMC5793797; doi:10.15252/embj.201797651)
Supplement: Supplementary file 1 — Appendix [file EMBJ-37-413-s001.pdf]

Appendix

for

OxyS small RNA induces cell cycle arrest to allow DNA damage repair

Shir Barshishat, Maya Elgrably-Weiss, Jonathan Edelstein, Jens Georg,

Sutharsan Govindarajan, Meytal Haviv, Patrick R. Wright, Wolfgang R. Hess, Shoshy Altuvia

Table of contents

Appendix figures

Appendix figure S1-S12

Appendix tables

Appendix table S1

Appendix table S2

Appendix table S3

Appendix references

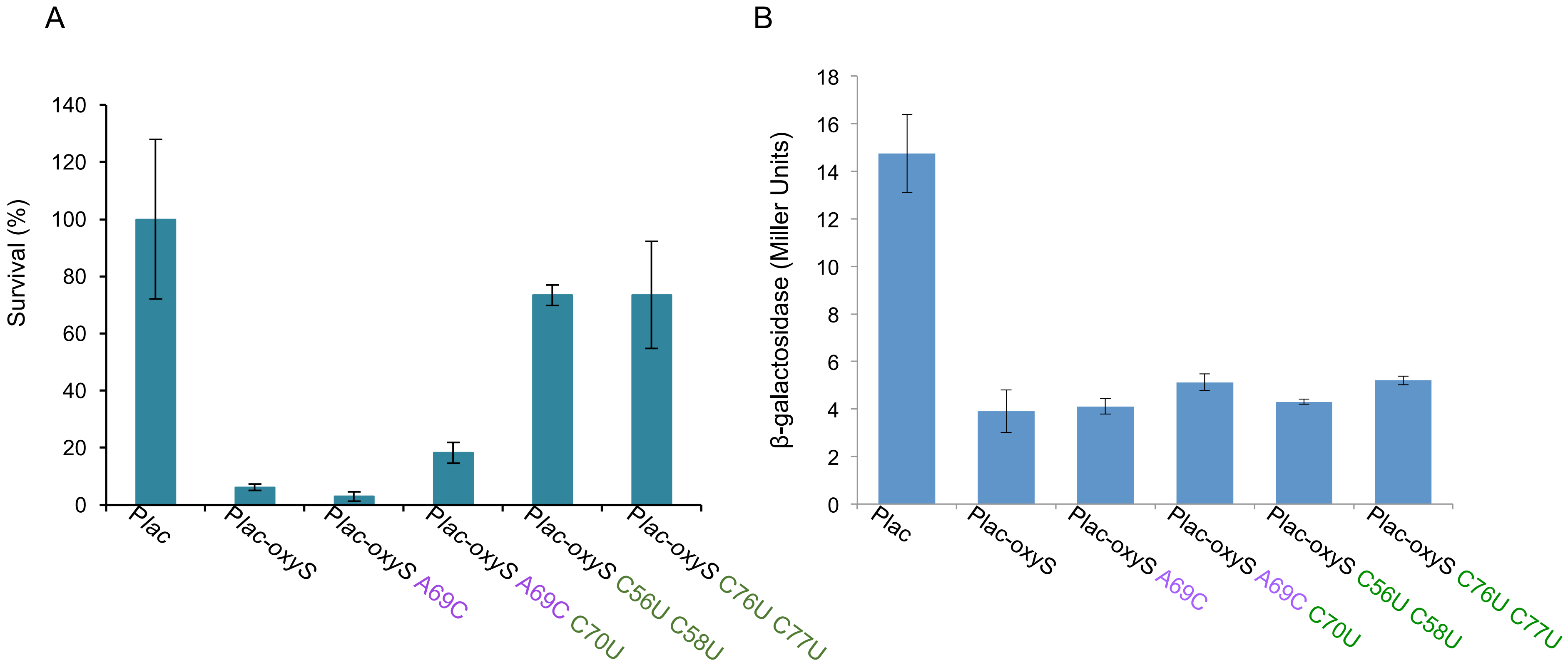

Appendix Figure S1. Characterization of OxyS toxic and nontoxic mutants. (**A**) Expression of OxyS is detrimental. Cultures (MG1655 *relA*::*cat*, *lacI*^{q}) carrying plasmids were treated with 0.4mM IPTG at OD600 of 0.1. Samples were taken prior to and 60 minutes after the addition of IPTG. Survival of cells carrying P*lac* plasmid was used as 100% reference. **(B)** *fhlA-lacZ* repression by OxyS toxic and nontoxic mutants. Cells carrying λ*fhlA-lacZ* translation fusion and OxyS plasmids were treated with 1mM IPTG for 1 hour at OD600 of 1. (MG1655 λ*fhlA-lacZ*: *kan*, *relA*::*cat*, *lacI*^{q}*,* *lacZ*::Tn10). Results are displayed as mean of 2 biological experiments ± standard deviation.

A

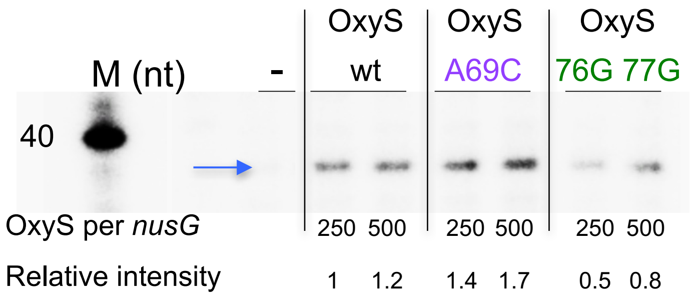

Appendix Figure S2A. RNase protection assay. OxyS RNAs, wild type and mutants were mixed with fully labeled *nusG* mRNA at the indicated ratios. After heating, the mixture was cooled and the RNAs were allowed to anneal at 45ºC. The mixture was then treated with single stranded specific ribonucleases (RNAse A and T1) to cleave unpaired nucleotides. The radioactive digestion products were separated using 8% urea-polyacrylamide sequencing gel. As basepairing between OxyS and *nusG* is discontinuous, annealing of *nusG* to OxyS mutant RNAs was estimated based on the formation of the full-length hybrid. OxyS-*nusG* full-length hybrid spans at least 35 nucleotides. Relative intensity denotes the ratio of the full-length hybrids obtained, compared to wild type *oxyS-nusG* interaction, which was used as a 100% reference. All lanes are from the same blot/exposure from which irrelevant interspersed lanes were removed.

B

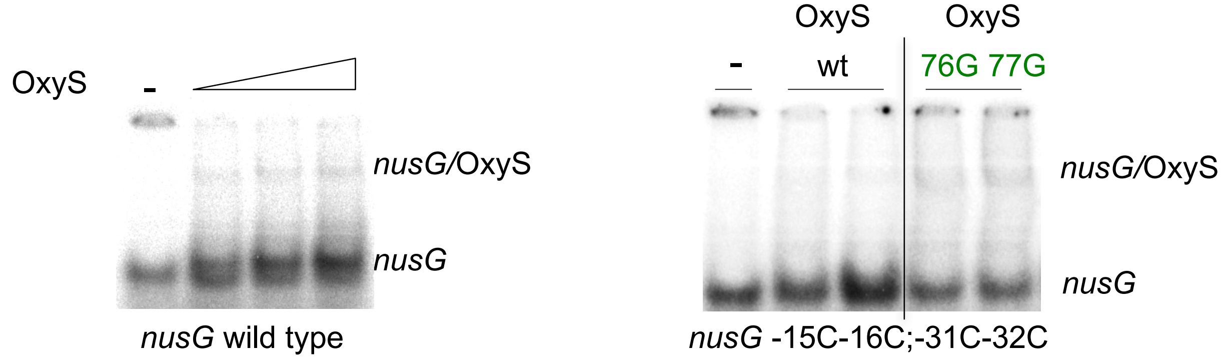

Appendix Figure S2B. EMSA. 0.01 pmol (left panel) and 0.004 pmol (right panel) of fully labeled wild type and quadruple mutant *nusG* mRNA (176 nt), respectively, without and with OxyS (left panel; 5, 10, 15 pmol and right panel 2, 4 pmol) were incubated in DEPC treated water at 85ºC for 15 min. Thereafter, the mixtures were incubated at 37ºC (left panel) or 42ºC (right panel) for 60 min in binding buffer as described in Materials and Methods. The RNAs were separated using 4.5 % polyacrylamide native gel. All lanes are from the same blot/exposure from which irrelevant interspersed lanes were removed.

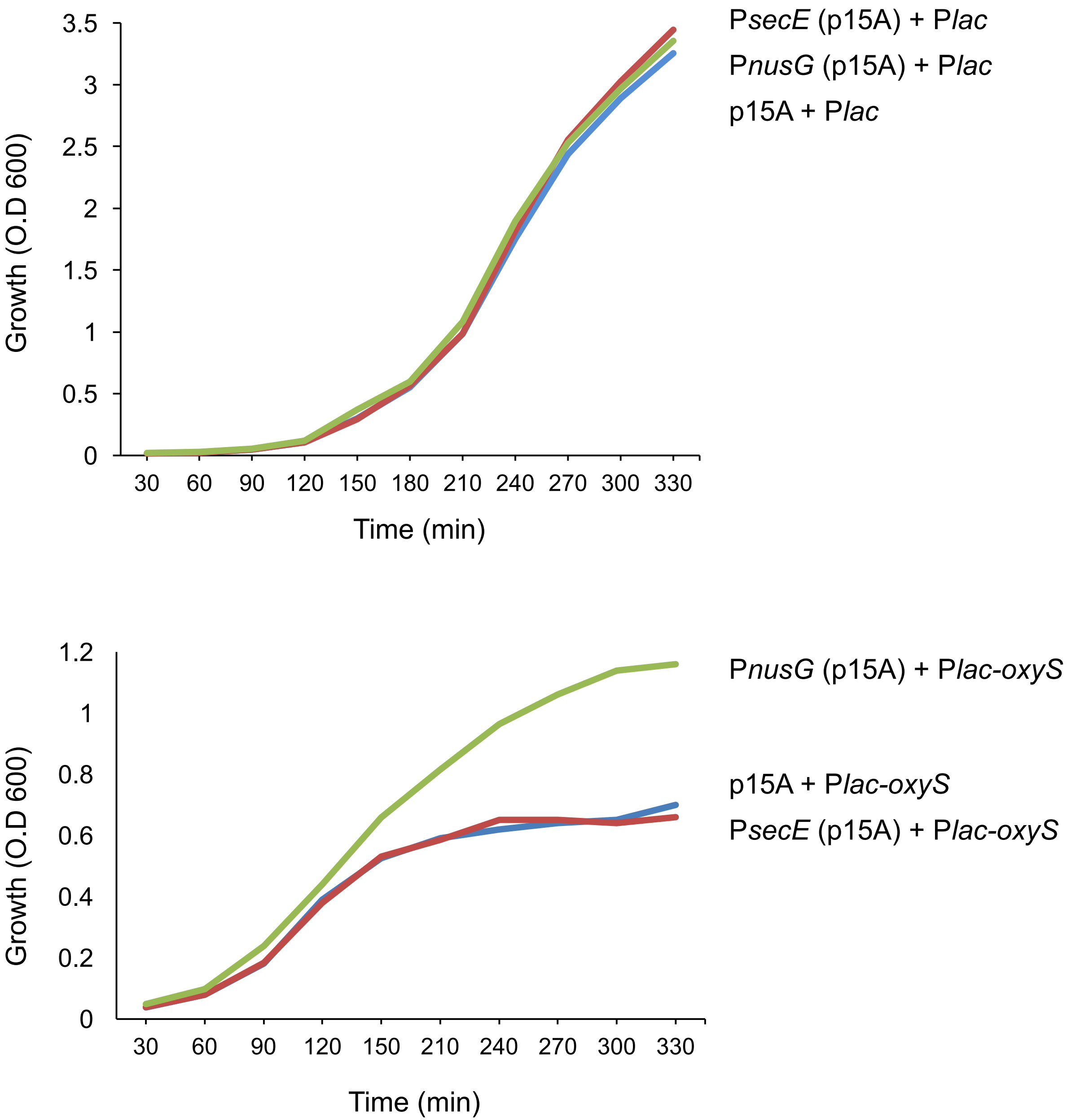

Appendix Figure S3. In *trans* expression of NusG (as opposed to SecE) rescues cells from the toxic effects of OxyS. Growth curves of cultures (MG1655 *relA*::*cat*, *lacI*^{q}) carrying control (top) or OxyS plasmids (bottom) were treated with 1mM IPTG at dilution.

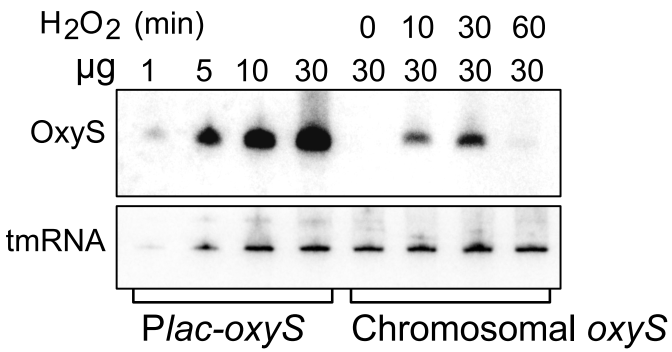

Appendix Figure S4. Over night cultures carrying the plasmid P*lac*-*oxyS* were diluted 1 to 100 in fresh medium supplemented with 1 mM of IPTG to induce expression of OxyS. Total RNA was collected after 90 of growth. Cultures carrying an intact chromosomally encoded *oxyS* allele were treated with 1 mM of hydrogen peroxide at OD600 of 0.1 for the indicated time points. The amounts of total RNA loaded are 1 to 30 µg as indicated. The membrane was probed with *oxyS* and tm specific primers.

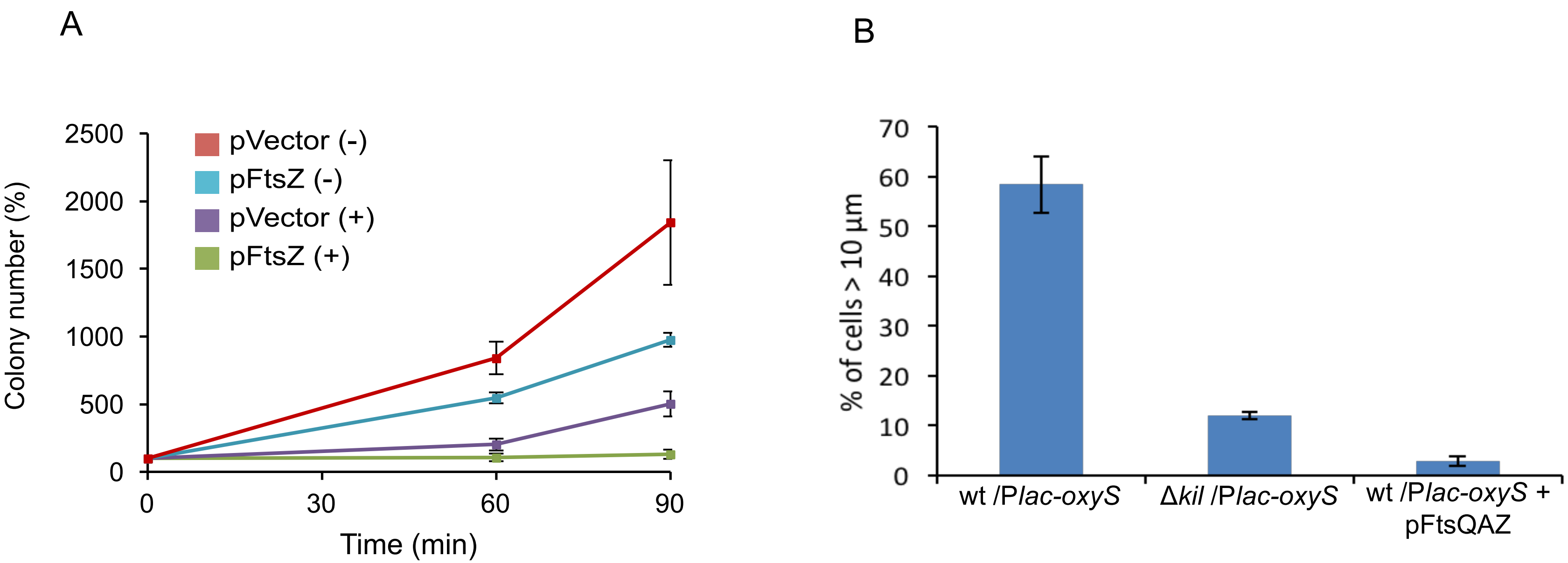

Appendix Figure S5. OxyS, KilR and FtsZ effect on recovery and cell length (**A**) OxyS mediated recovery requires impaired FtsZ. Cultures carrying the control plasmid (pVector) and pFtsQAZ (pFtsZ) were grown to OD600 of 0.1 at which half of each culture was exposed to 1mM H2O2 for 30 min (+). Thereafter, the cultures were washed and grown as described in Figure 4. Results are displayed as mean of 4 biological experiments ± standard deviation. **(B)**. Cell length distribution of cultures expressing OxyS (P*lac-oxyS*) in wild type and in Δ*kilR* mutant or of wild type cultures expressing OxyS and pFtsQAZ (pFtsZ). Results are displayed as mean of 3 biological experiments of each culture ± standard deviation. The total number of cells analyzed for wt/p*lac-oxyS*, Δ*kilR*/p*lac-oxyS* and wt/*plac-oxyS* + pFtsZ were 884, 1010 and 1109, respectively. Statistics (unpaired t-test) was carried out using GraphPad prism software. For all the data the p-value is <0.0001. The cultures were grown as described in Figure 5.

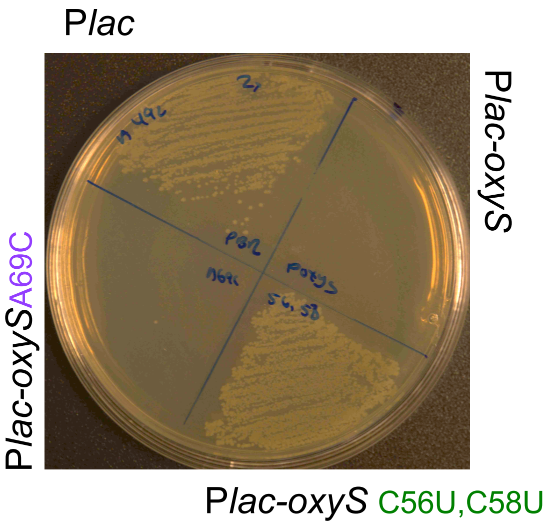

Appendix Figure S6. RW434 *(lexA3* SOS-off) were transformed with control and OxyS expressing plasmids. OxyS is still toxic in SOS off cells.

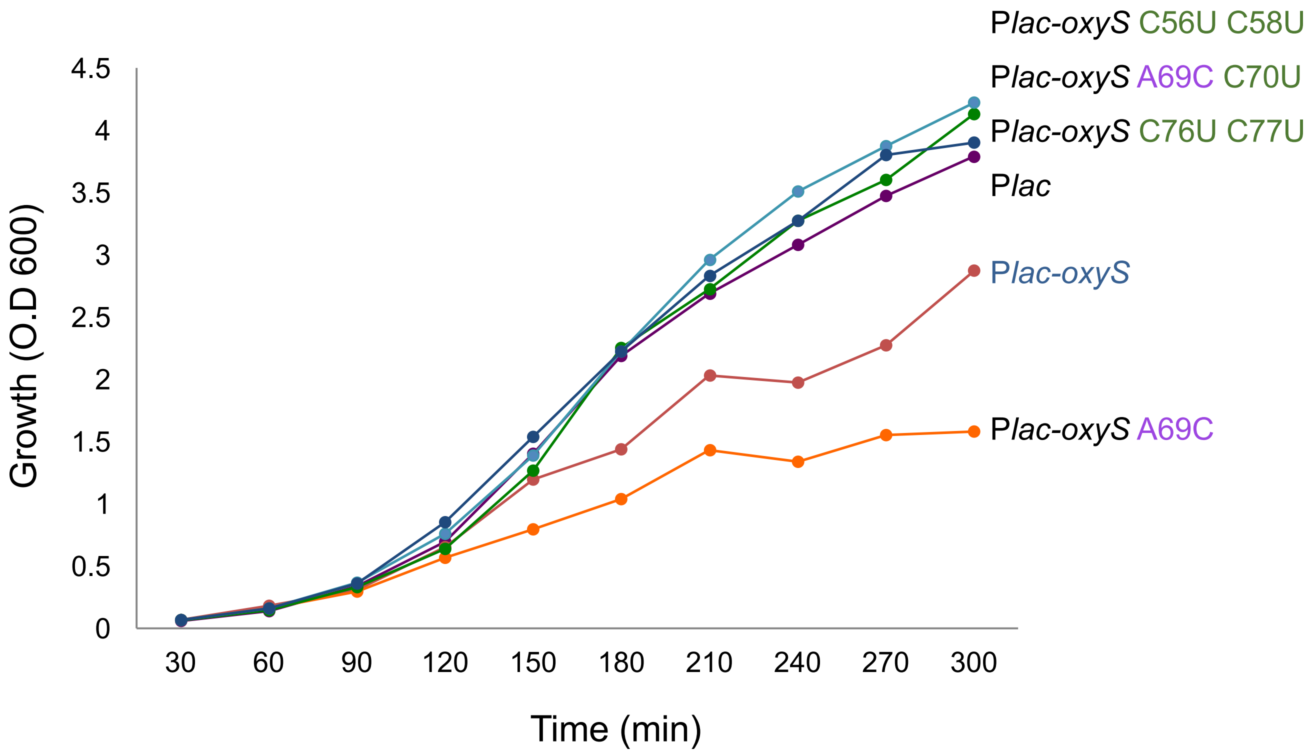

Appendix Figure S7. Growth curves of cells carrying a wild type *relA* allele and OxyS plasmids. Cultures carrying plasmids were treated with 1mM IPTG at dilution. O.D was measured as indicated. Suppressor nontoxic mutants (green) highly toxic mutant (purple) (MG1655 *lacI*^{q}).

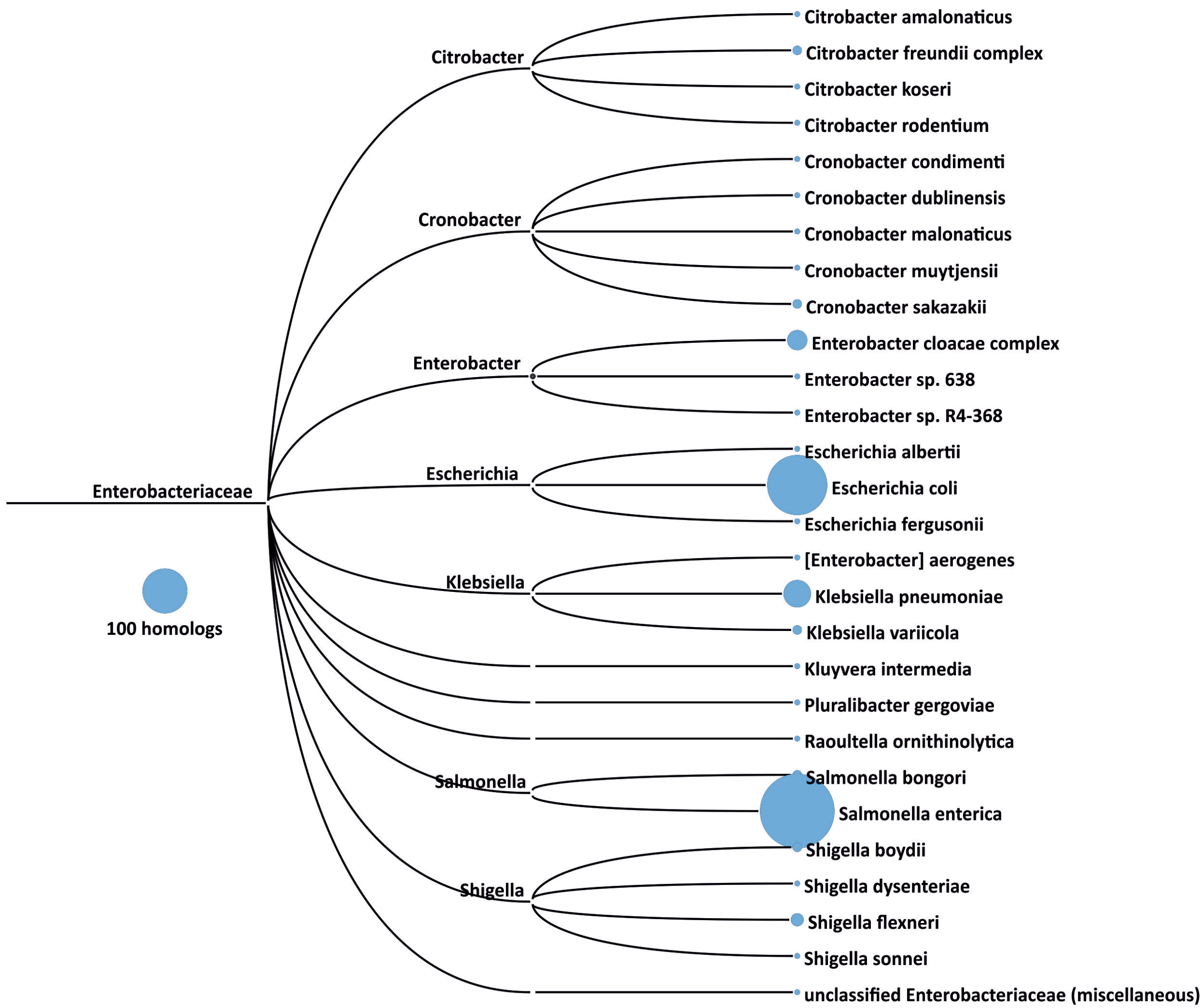

Appendix Figure S8. Taxonomic tree of 603 OxyS homologs. OxyS homology search was based on iterative blasting and structural filtering implemented in the GLASSgo webserver (http://rna.informatik.uni-freiburg.de/GLASSgo/Input.jsp). The tree was generated with Megan (Huson *et al*, 2016). The area of the blue circles indicates the number of detected homologs per genus.

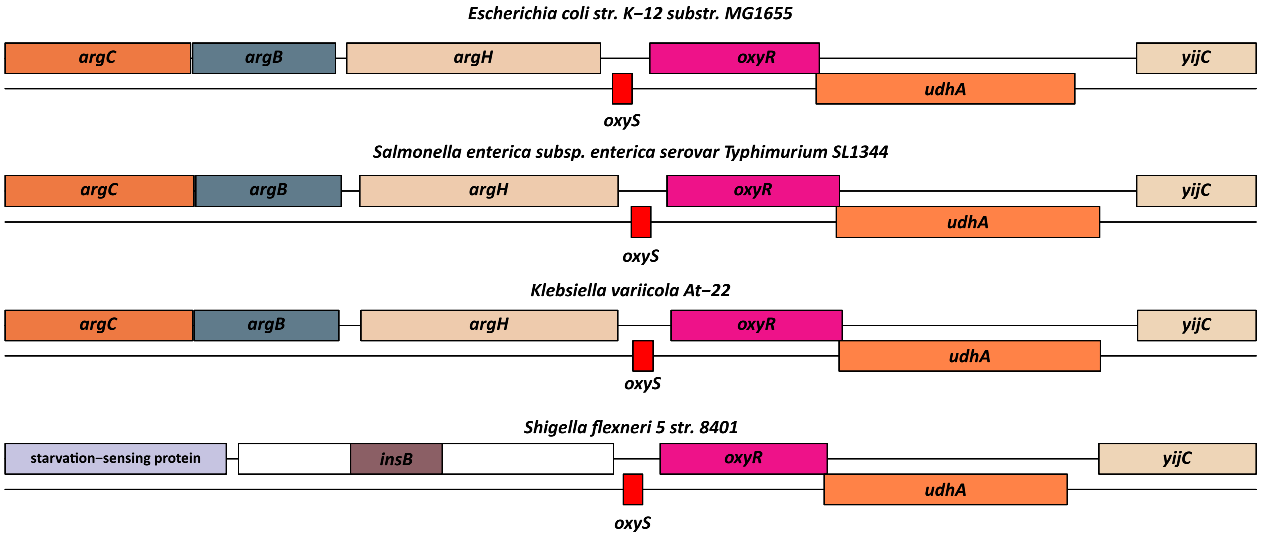

Appendix Figure S9. Examples for the conserved synteny around *oxyS*. The loci of OxyS genes in several species are depicted schematically. All homologs are located in the 5' intergenic region of *oxyR* and share a widely conserved synteny.

A

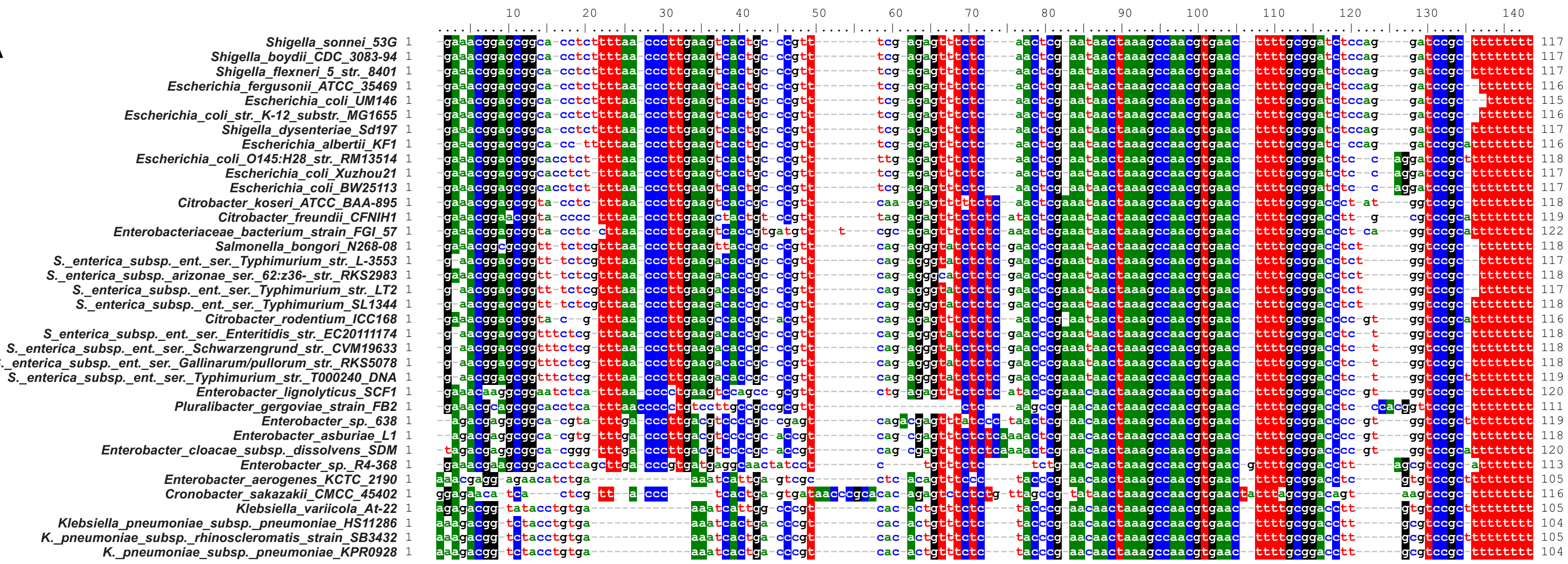

B

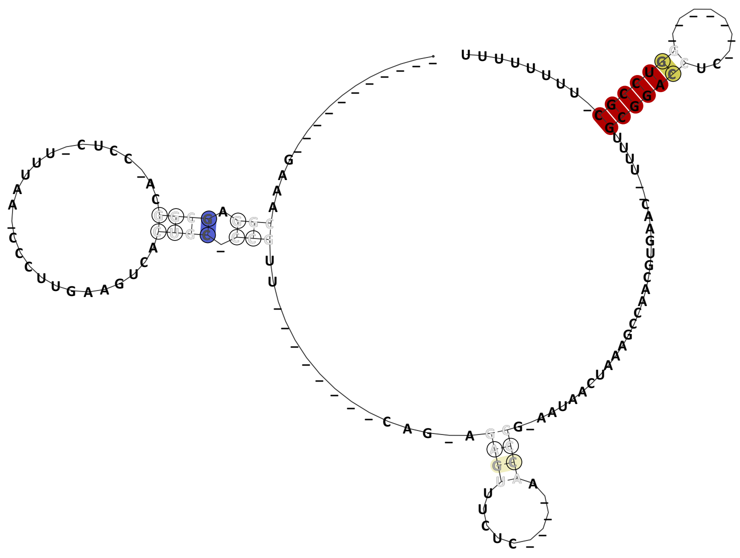

Appendix Figure S10. Multiple sequence-structure alignment of 36 OxyS homologs carried out with MAFFT Q-INS-i (Katoh & Toh, 2008). (**A**) Bases conserved ≥ 90% are highlighted. **(B)** OxyS consensus secondary structure generated by RNAalifold (Bernhart *et al*, 2008) based on the alignment in A.

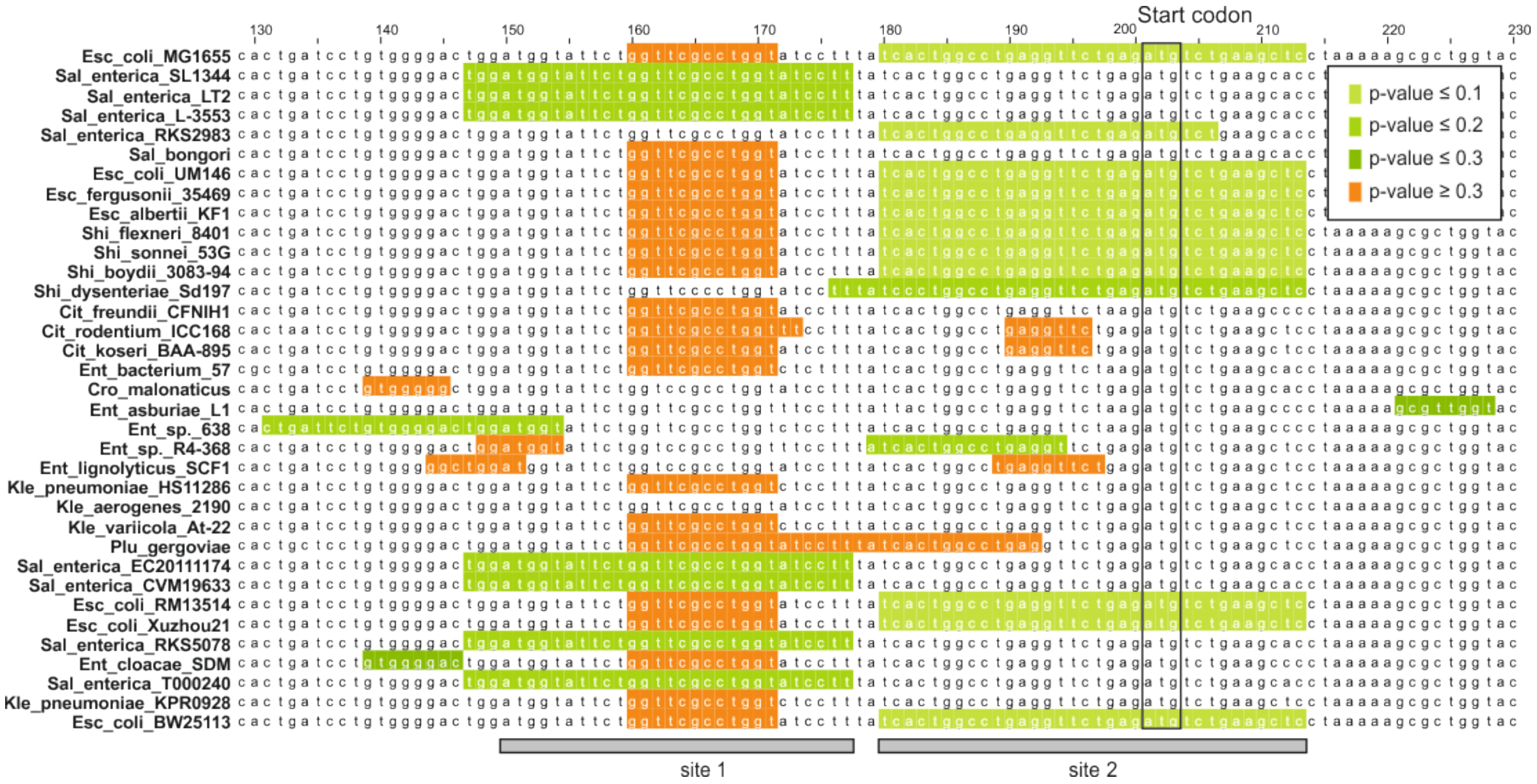

Appendix Figure S11. Alignment of the 5'UTRs of the 35 investigated *nusG* homologs. The start codon of NusG is marked by a black box. The interaction regions referring to the optimal and the first suboptimal organism specific predictions from the IntaRNA output with "Turner 1999" energy parameters (Wright *et al*, 2013) are marked by a colored background. The color indicates the respective prediction p-value with shades of green for a p-value ≤ 0.3 and orange for p-values > 0.3. Two prominent predicted interaction site regions are indicated below the alignment by grey boxes. Site 1 binds upstream of *nusG* ribosome binding site while site 2 overlaps the *nusG* start codon and/or the *nusG* SD region.

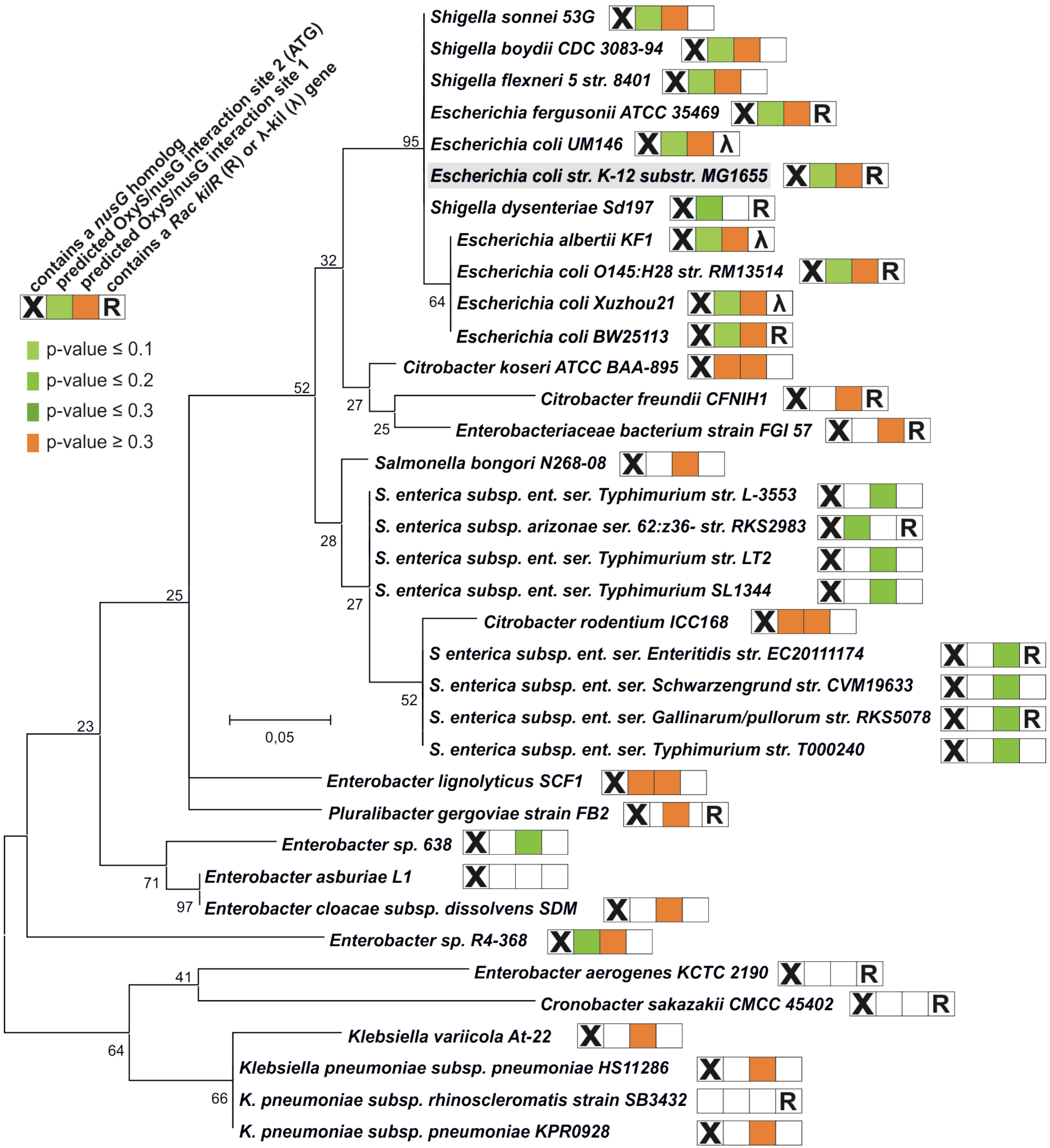

Appendix Figure S12. Maximum likelihood tree based on the multiple OxyS alignment above. A "X" in the first square indicates existence of a *nusG* homolog, the second square indicates a predicted OxyS/*nusG* hybrid overlapping the ribosome binding site (site2 regarding to alignment figure), the third square indicates a predicted OxyS/*nusG* hybrid located ~20 nt 5' of *nusG* start codon (site1 regarding to alignment figure). The color in square two and three indicates the respective prediction p-value with shades of green for a p-value ≤ 0.3 and orange for p-values > 0.3, white indicates no prediction at this position. The fourth square indicates the existence of a *kilR* (R) or a λ-kil (λ) homolog . The interaction regions refer to the optimal and the first suboptimal organism specific predictions from the IntaRNA output with "Turner 1999" energy parameters (Wright *et al*, 2013). Conservation of *nusG* and *kilR* was analyzed using blastp with standard parameters (E-value ≤ 10, word size 6, BLOSUM62) against the individual genomes. The evolutionary history was inferred by using the Maximum Likelihood method based on the (Tamura & Nei, 1993). The tree with the highest log likelihood (-557,6917) is shown. The percentage of trees in which the associated taxa clustered together is shown next to the branches. Initial tree(s) for the heuristic search were obtained automatically by applying Neighbor-Joining and BioNJ algorithms to a matrix of pairwise distances estimated using the Maximum Composite Likelihood (MCL) approach, and then selecting the topology with superior log likelihood value. The tree is drawn to scale, with branch lengths measured in the number of substitutions per site. The analysis involved 36 nucleotide sequences. All positions containing gaps and missing data were eliminated. There were a total of 76 positions in the final dataset. Evolutionary analyses were conducted in MEGA7 (Kumar *et al*, 2016).

Appendix Tables

Appendix Table S1. Strains

Lab stock	Relevant genotype[a]	Source or reference
A1	MG1655 *E. coli K-12 F- lambda- ilvG- rfb-50 rph-1*	Lab collection
A2	MC4100 *E. coli F- araD139* Δ*(argF-lac)U169 rpsL150 relA1 flbB5301 deoC1 ptsF25 rbsR*	Lab collection
A402	MC4100 *relA*::*cat*	Lab collection
A411	DY378 W3110 λcI857 Δ(*cro-bioA*)	(Yu *et al*, 2000)
A438	MC4100 *relA*::*kan*	Lab collection
A492	RW434	(Fernandez De Henestrosa *et al*, 2000)
A708	MG1655 *mal*::*lacI*^{q}	(Guillier & Gottesman, 2006)
A782	(A708) *mal*::*lacI*^{q} Δ*relA*::*cat* (of A-402)	This study
A807	MDS42 MG1655 Δ*fhuACDB* Δ*endA* and deletion of 699 additional genes, including all IS elements and cryptic prophages	Lab collection
A817	(A708) *mal*::*lacI*^{q} Δ*lacZ*::*Tn*10	This study
D60	MC4100 λRS552-*fhlA-lacZ*	(Altuvia *et al*, 1998)
D999	(A817) *mal*::*lacI*^{q} Δ*lacZ*::*Tn10* λRS552-*fhlA-lacZ* (of D60)	This study
A838	(A708) *mal*::*lacI*^{q} Δ*oxySli*::*kan*	This study
A840	(A782) *mal*::*lacI*^{q} Δ*relA*::*cat* Δ*oxySli*::*kan*	This study
A843	(A838) *mal*::*lacI*^{q} Δ*oxySli*::*frt*	This study
A850	(A782) *mal*::*lacI*^{q} Δ*relA*::*cat* Δ*oxySli*::*frt*	This study
A854	(A807) MDS42 *nusG*-SPA:*kan*	This study

A855	(A838) *mal*::*lacI*^{q} Δ*relA*::*cat* Δ*oxySli*::*frt lacZ*::*Tn*10	This study
A863	(A782) *mal*::*lacI*^{q} Δ*relA*::*cat nusG*-SPA *kan* (of A-854)	This study
A870	(A708) *mal*::*lacI*^{q} Δ*kilR*::*cat*	This study
A872	(A708) *mal*::*lacI*^{q} Δ*relA*::*kan* (of A-438)	This study
A873	(A870) *mal*::*lacI*^{q} Δ*kilR*::*cat* Δ*relA*::*kan* (of A-438)	This study
A893	(A843) *mal*::*lacI*^{q} Δ*relA*::*kan* (of A-438) Δ*oxySli*::*frt*	This study
A894	MG1655 Δ*lacIZYA*::*frt zapA-gfp*:*cat*	(Peters *et al*, 2011)
A897	(A872) *mal*::*lacI*^{q} Δ*relA*::*kan zapA-gfp*:*cat* (of A894)	This study
A920	(A893) *mal*::*lacI*^{q} Δ*relA*::*kan* (of A-438) Δ*oxySli*::*frt* Δ*kilR*::*cat*	This study
A983	(A840) *mal*::*lacI*^{q} Δ*relA*::*cat* Δ*oxySli*::*kan nusG*-SPA *kan* (of A-854)	This study

[a]Chromosomal gene mutants and gene fusions were transferred by P1 transduction (see for example of A854)

Appendix Table S2. Plasmids

**Plasmids**	**Genetic elements**	**Origin**	**Marker**	**Source or reference**
pKK177-3	P*tac*	ColE1	Amp^r	(Altuvia *et al*, 1997)
pJL148			Kan^r	(Zeghouf *et al*, 2004)
poxyS	P*tac-oxyS*	ColE1	Amp^r	(Altuvia *et al*, 1997)
poxyS$_{A69C}$	P*tac-oxyS*$_{A69C}$	ColE1	Amp^r	Lab collection
pBR-P*lac*	P*lacO*	ColE1	Amp^r	(Guillier & Gottesman, 2006)
pSA84	P*lacO-oxyS*	ColE1	Amp^r	This study
pSA85	P*lacO-oxyS*$_{A69C}$	ColE1	Amp^r	This study
pSA86	P*lacO-oxyS*$_{AC69,70CU}$	ColE1	Amp^r	This study

pSA87	P*lacO-oxyS*C56U,C58U	ColE1	Amp^r	This study
pSA88	P*lacO-oxyS*CC76,77UU	ColE1	Amp^r	This study
pSA89	P*lacO-oxyS*CC76,77GG	ColE1	Amp^r	This study
pACYC184		p15A	Cm^r Tet^r	Lab collection
pFtsQAZ		pSC101	Tet^r	(Bernhardt & de Boer, 2004)
pSA68	*lacZ* (translation fusion)	pSC101*	Kan^r	(Hershko-Shalev *et al*, 2016)
pSA90	P*tac-nusG-lacZ*	pSC101*	Kan^r	This study
pSA91	P*tac-nusG*C-15G,C-16G-*lacZ*	pSC101*	Kan^r	This study
pSA92	P*tac-nusG*C-31G,C-32G-*lacZ*	pSC101*	Kan^r	This study
pSA93	P*tac-nusG*C-15G,C-16G,C-31G,C-32G-*lacZ*	pSC101*	Kan^r	This study
pSA94	(B1379) pACYC184 P_{secE} *secE-nusG*	p15A	Cm^r	This study
pSA95	(B1518) pACYC184 P_{secE} *secE*	p15A	Cm^r	This study
pSA96	(B1519) pACYC184 P_{secE} *nusG*	p15A	Cm^r	This study
pSA97	(B1511) P*lacO-kilR*	ColE1	Amp^r	This study

Appendix Table S3. Oligonucleotides

Oligonucleotides used for cloning

**Primer**	**Primer sequence (5'-3')**	**Use**[a]
2026	CCC**GACGTC**GAAACGGAGCGGCACCTC	*oxyS* (AatII +)
2027	CCC**AAGCTT**ATCGCCGGCTT TTTATGG	*oxyS* (HindIII -)
2179	G**GAATTC**GCCGTAGTAATTCTGATTGC	*nusG* (EcoRI +)
2181	TCC**CCCGGG**GGTTGGTACCATGACTTCACC	*nusG* (SmaI -)

2377	CCC**AAGCTT**CTGACGTGACTGGTACC	*secE-nusG* (HindIII +)
2378	CG**GGATCC**GCGCGAAATTGTATTCC	*secE-nusG* (BamHI -)
2700	CCC**GACGTC**TTGTAAAAATGGAGATAATTATGATTGC	*kilR* (AatII +)
2701	CCC**AAGCTT**TGCAAAGGTGGTAAGCAC	*kilR* (HindIII -)
2725	CTGTGGGGACTGGATGG	*nusG* (+)
2726	CGCACTCATAAACCAACC	*nusG* (-)
2727	CG**GGATCC**CTGAACGACGTACCAGC	*secE* (BamHI -)

[a]Plus (+) and minus (-) strands are indicated. The restriction sites are denoted in bold

Oligonucleotides used for Real-Time PCR

Primer	Primer sequence (5'-3')	Use[a]
1309	CAGAGATGAGAATGTGCCTTCGGG	*rrsB* (+)
1310	CC CTGGCAACAAAGGATAAGG	*rrsB* (-)
2531	CCACGTCAGTGTGTGACTC	*kilR* (+)
2532	CCGCCTTTACAGGTAAACC	*kilR* (-)

[a]Plus (+) and minus (-) strands are indicated

Oligonucleotides used for construction of strains

Primer	Primer sequence (5'-3')	Use[a]
2081	ATGACCATGATTACGGATTCACTGGCCGTCGTTACTCGACATCTTGGTTACCG	Δ*lacZ* (*tet* +)
2082	TTATTTTTGACACCAGACCAACTGGTAATGGTAGCAAGAGGGTCATTATATTTCG	Δ*lacZ* (*tet* -)
2083	CCCAGGCTTTACACTTTATGC	Δ*lacZ*
2084	CATAATGGATTTCCTTACGCG	Δ*lacZ*

2169	CCTCTTTTAACCCTTGAAGTCACTGCCCGTTTCTGTAGGCTGGAGCTGCTTC	Δ*oxySli* (*kan* +)
2170	TATGGCAAAAAAAAGCGGATCCTGGAGATCCGCATGGGAATTAGCCATGGTCC	Δ*oxySli* (*kan* -)
2265	CAGACACCGCATCAACAAAGTTCATTTGTAAAAATGGAGATGGGAATTAGCCATGGTCC	Δ*kilR* (*cat* +)
2295	TAAAATCCCGTGCATGAAGTGAAGCATTCTTTCAATAGCTGTAGGCTGGAGCTGCTTC	Δ*kilR* (*cat* -)
2267	CTGGTTGCAGTCACCTG	Δ*kilR*
2296	TTCATGTGCCATCTGGTC	Δ*kilR*
2216	GCGACCCCGGTAGAGCTGGACTTCAGCCAGGTTGAAAAAGCCTCCATGGAAAAGAGAAG	*nusG*-SPA (+)
2217	ACGCCTTGTGCAACGATTAAATCGCCGCTTTTTTGATCGCTGGGTTAGTTCCTATTCCGAAGTTC	*nusG*-SPA (-)
2182	GTGAAGTCATGGTACCAAC	*nusG*-SPA insertion (+)
2219	TCCAATCTCACGCCT TG	*nusG*-SPA insertion (-)
2227	GGATGAGATTTTCTTAAAGCGG	SPA sequence

[a]Plus (+) and minus (-) strands are indicated. The underlined bases are complementary to the specific cassette used

Oligonucleotides used for site-directed mutagenesis

Primer	Primer sequence (5'-3')	Use[a]
2039	CCAACGTGAACTTTTGC	$A_{69}C$ (+), $AC_{69,70}CU$ (+)
2041	CTTTAG**G**TATTCGAGTTGAGAAAC	*oxyS* $A_{69}C$ (-)
2040	CTTTA**AG**TATTCGAGTTGAGAAAC	*oxyS* $AC_{69,70}CU$ (-)
2079	GTTT**T**T**T**AACTCGAATAACTAAAGCC	*oxyS* $C_{56}U$, $C_{58}U$ (+)
2080	TCTCGAAACGGGCAGTG	*oxyS* $C_{56}U$, $C_{58}U$ (-)
2037	**TT**AACGTGAACTTTTGC	*oxyS* $CC_{76,77}UU$ (+)
2038	CTTTAGTTATTCGAGTTGAG	*oxyS* $CC_{76,77}UU$, $CC_{76,77}GG$ (-)
2408	**GG**AACGTGAACTTTTGCG	*oxyS* $CC_{76,77}GG$ (+)

2409	**CC**CCTGAGGTTCTGAGATG	*nusG* GG$_{-15-16}$CC (+)
2410	AGTGATAAAGGATACCAGGC	*nusG* GG$_{-15-16}$CC (-)
2707	**CC**TATCCTTTATCACT**CC**CCTGAGGTTCTGAGATG	*nusG* quadruple (+)
2709	AGGCGAACCAGAATACCATC	*nusG* quadruple (-)
2738	**CC**TATCCTTTATCACTGGCC	*nusG* GG$_{-31-32}$CC (+)
2739	AGGCGAACCAGAATACC	*nusG* GG$_{-31-32}$CC (-)

[a]Plus (+) and minus (-) strands are indicated. The mutations are denoted in bold

Oligonucleotides used for *in vitro* RNA synthesis

Primer	Primer sequence (5'-3')	fragment[a]
2220	**CGAAATTAATACGACTCACTATAGGGACAGG**GTAATGTCACTGATCCTG	**PT7**- *nusG* (+)
2221	GTAATTTGATATGCTCACG	*nusG* (-)
2238	**CGAAATTAATACGACTCACTATAGGGACAGG**GAAACGGAGCGGCACC	**PT7**- *OxyS* (+)
2620	**CGAAATTAATACGACTCACTATAGGGACAGG**GTAATTTGATATGCTCACG	**PT7**- *anti nusG* (-)
2554	AGTATTCTGGTTCGCCTG	*anti nusG* (+)

[a]Plus (+) and minus (-) strands are indicated. The nucleotides in bold denote the sequence of the T7 promoter (**PT7**)

Oligonucleotides used for northern

Primer	Primer sequence (5'-3')	Use
492	GCGGATCCTGGA	*oxyS*
1912	CCGCGTCCGAAATTCCTA	tmRNA

Appendix references

Altuvia S, Weinstein-Fischer D, Zhang A, Postow L, Storz G (1997) A small, stable RNA induced by oxidative stress: role as a pleiotropic regulator and antimutator. *Cell* 90: 43-53

Altuvia S, Zhang A, Argaman L, Tiwari A, Storz G (1998) The *Escherichia coli* OxyS regulatory RNA represses *fhlA* translation by blocking ribosome binding. *Embo J* 17: 6069-75

Bernhardt TG, de Boer PA (2004) Screening for synthetic lethal mutants in *Escherichia coli* and identification of EnvC (YibP) as a periplasmic septal ring factor with murein hydrolase activity. *Mol Microbiol* 52: 1255-69

Bernhart SH, Hofacker IL, Will S, Gruber AR, Stadler PF (2008) RNAalifold: improved consensus structure prediction for RNA alignments. *BMC Bioinformatics* 9: 474

Fernandez De Henestrosa AR, Ogi T, Aoyagi S, Chafin D, Hayes JJ, Ohmori H, Woodgate R (2000) Identification of additional genes belonging to the LexA regulon in *Escherichia coli*. *Mol Microbiol* 35: 1560-72

Guillier M, Gottesman S (2006) Remodelling of the *Escherichia coli* outer membrane by two small regulatory RNAs. *Mol Microbiol* 59: 231-47

Hershko-Shalev T, Odenheimer-Bergman A, Elgrably-Weiss M, Ben-Zvi T, Govindarajan S, Seri H, Papenfort K, Vogel J, Altuvia S (2016) Gifsy-1 Prophage IsrK with Dual Function as Small and Messenger RNA Modulates Vital Bacterial Machineries. *PLoS Genet* 12: e1005975

Huson DH, Beier S, Flade I, Gorska A, El-Hadidi M, Mitra S, Ruscheweyh HJ, Tappu R (2016) MEGAN Community Edition - Interactive Exploration and Analysis of Large-Scale Microbiome Sequencing Data. *PLoS Comput Biol* 12: e1004957

Katoh K, Toh H (2008) Improved accuracy of multiple ncRNA alignment by incorporating structural information into a MAFFT-based framework. *BMC Bioinformatics* 9: 212

Kumar S, Stecher G, Tamura K (2016) MEGA7: Molecular Evolutionary Genetics Analysis Version 7.0 for Bigger Datasets. *Mol Biol Evol* 33: 1870-4

Peters NT, Dinh T, Bernhardt TG (2011) A fail-safe mechanism in the septal ring assembly pathway generated by the sequential recruitment of cell separation amidases and their activators. *J Bacteriol* 193: 4973-83

Tamura K, Nei M (1993) Estimation of the number of nucleotide substitutions in the control region of mitochondrial DNA in humans and chimpanzees. *Mol Biol Evol* 10: 512-26

Yu D, Ellis HM, Lee EC, Jenkins NA, Copeland NG, Court DL (2000) An efficient recombination system for chromosome engineering in *Escherichia coli*. *Proc Natl Acad Sci U S A* 97: 5978-83

Wright PR, Richter AS, Papenfort K, Mann M, Vogel J, Hess WR, Backofen R, Georg J (2013) Comparative genomics boosts target prediction for bacterial small RNAs. *Proc Natl Acad Sci U S A* 110: E3487-96

Zeghouf M, Li J, Butland G, Borkowska A, Canadien V, Richards D, Beattie B, Emili A, Greenblatt JF (2004) Sequential Peptide Affinity (SPA) system for the identification of mammalian and bacterial protein complexes. *J Proteome Res* 3: 463-8
