# Supplementary figures and images for: OxyS small RNA induces cell cycle arrest to allow DNA damage repair
Source: EMBO J. 2017 Dec 13;37(3):413–26. doi: 10.15252/embj.201797651 (PMC5793797; doi:10.15252/embj.201797651)

# Source data for Appendix Fig. S2A

Sequencing gel 8% acrylamide

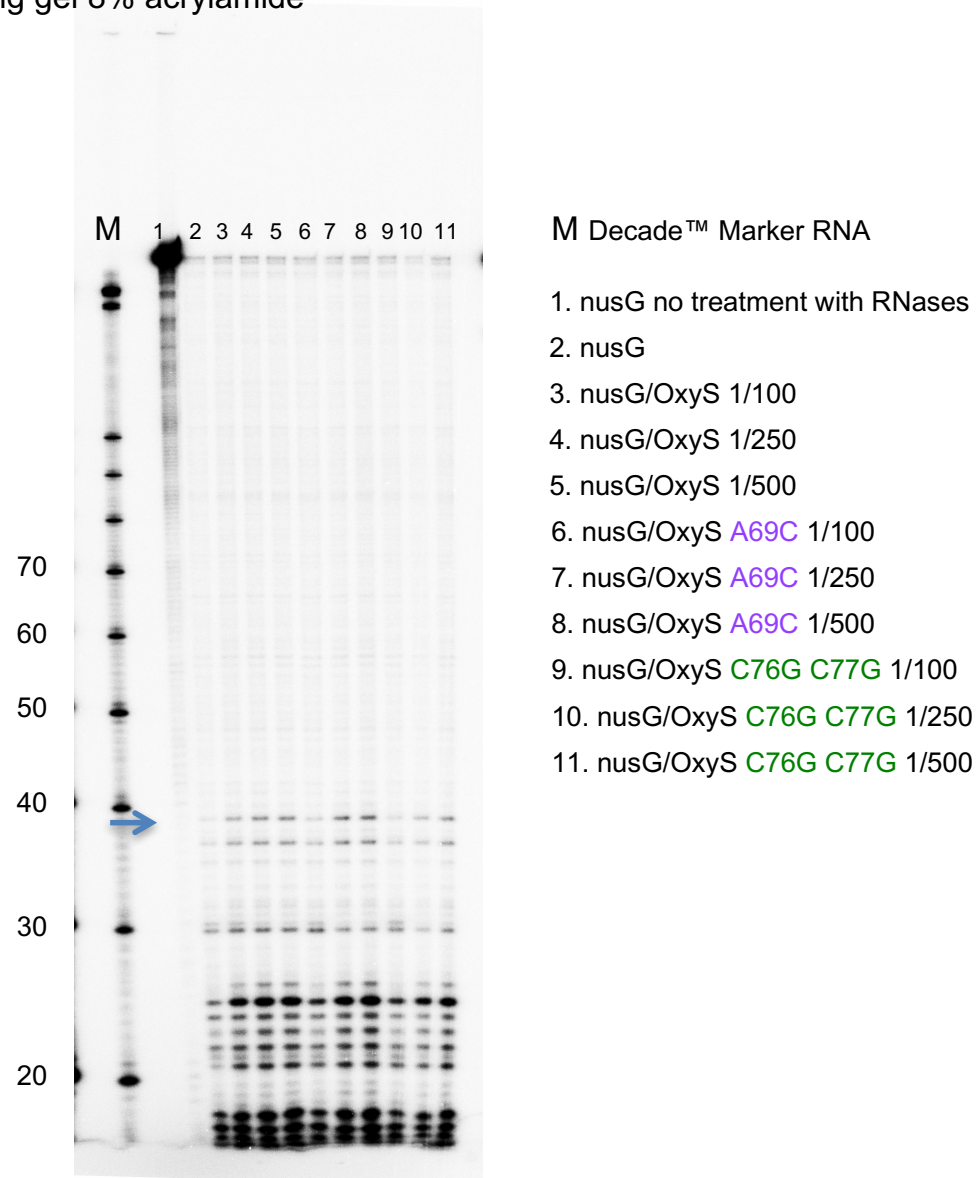

Supplement: Supplementary file 2 — Source Data for Appendix [file EMBJ-37-413-s004.zip › 97651_Source_Data_Fig_S2A.pdf]

## Source data for Appendix Fig. S2B

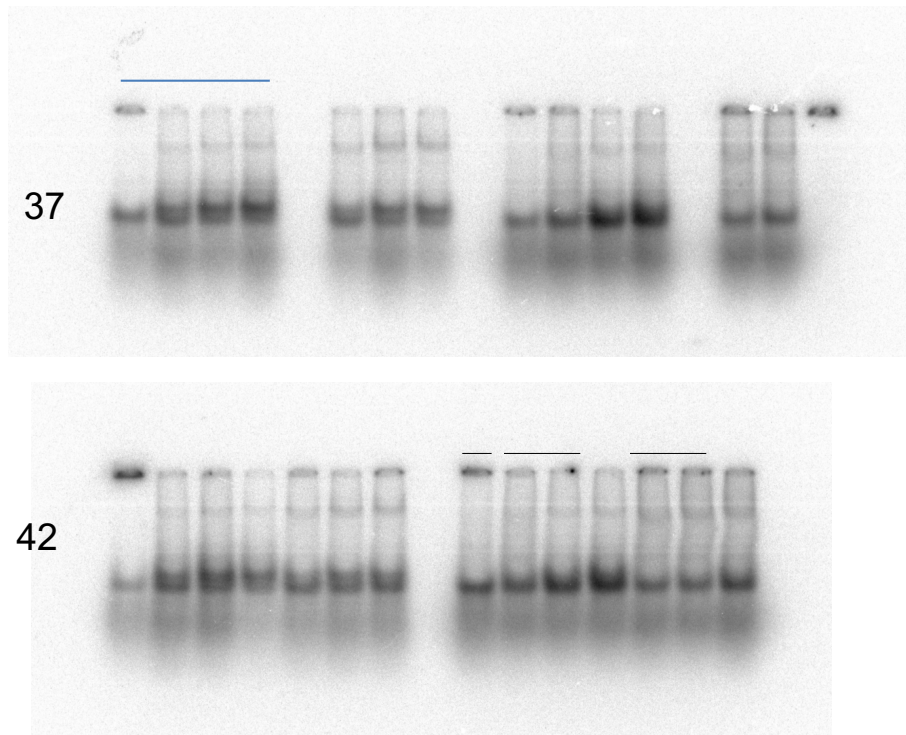

Supplement: Supplementary file 2 — Source Data for Appendix [file EMBJ-37-413-s004.zip › 97651_Source_Data_Fig_S2B.pdf]

## Source data for Appendix Fig. S4

Northern 6% poly acrylamide

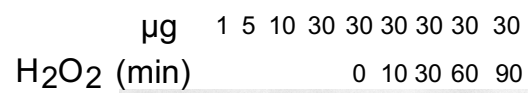

OxyS

μg 1 5 10 30 30 30 30 30 30

tm

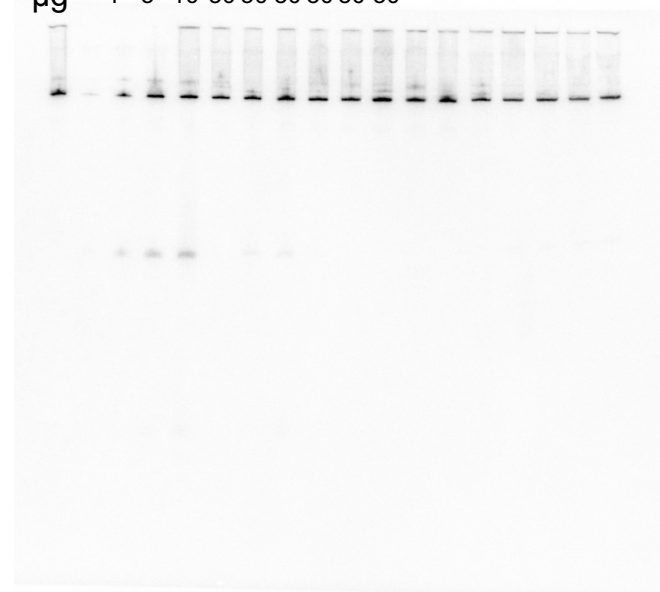

Supplement: Supplementary file 2 — Source Data for Appendix [file EMBJ-37-413-s004.zip › 97651_Source_Data_Fig_S4.pdf]

## Source data for Fig. 2B

Northern agarose gel 1.4%

15 µg total RNA

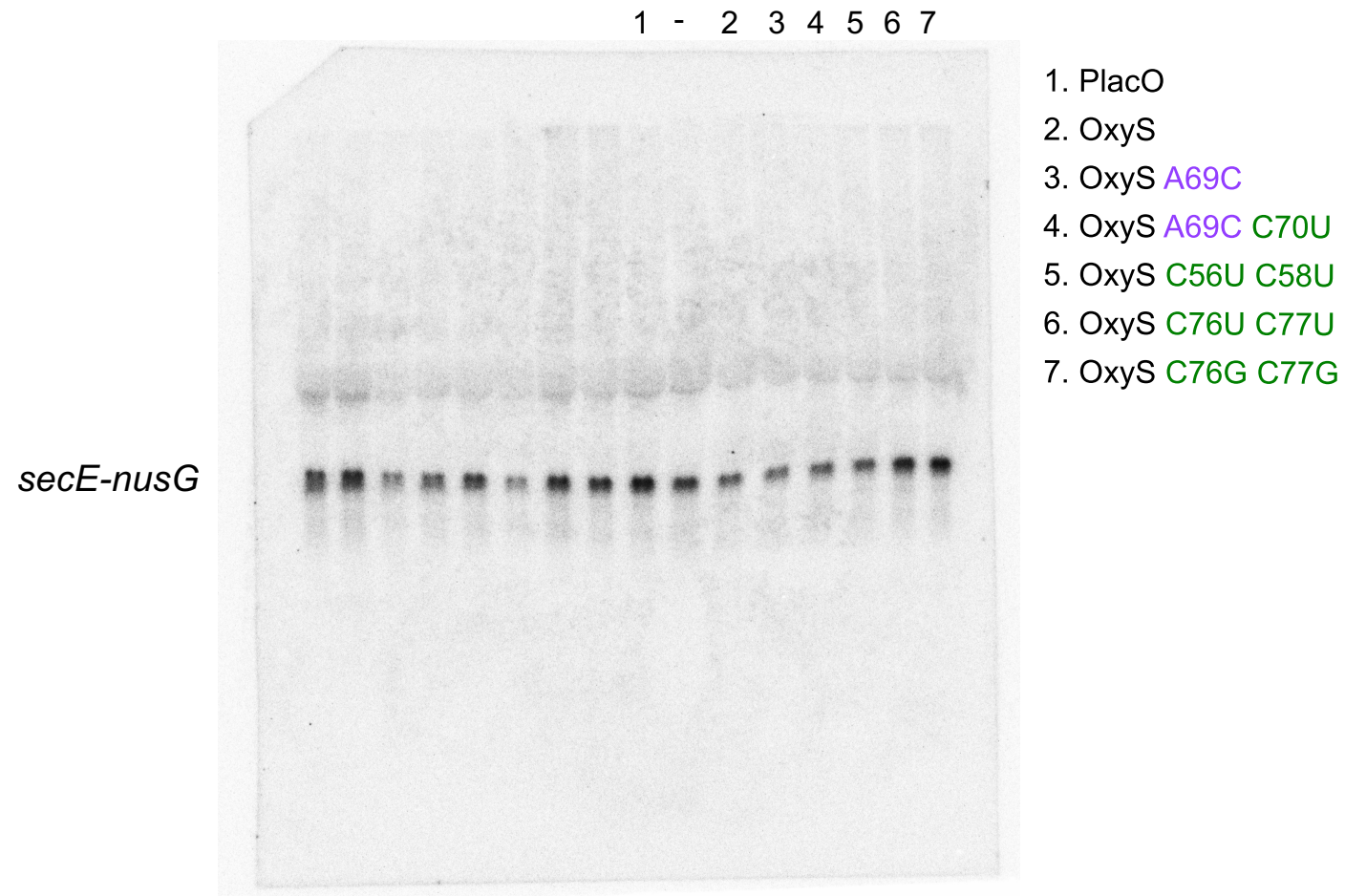

Supplement: Supplementary file 4 — Source Data for Figure 2 [file EMBJ-37-413-s002.zip › 97651_Source_Data_Fig_2B_SecENusG.pdf]

## Source data for Fig. 4D

Northern agarose gel 1.4%

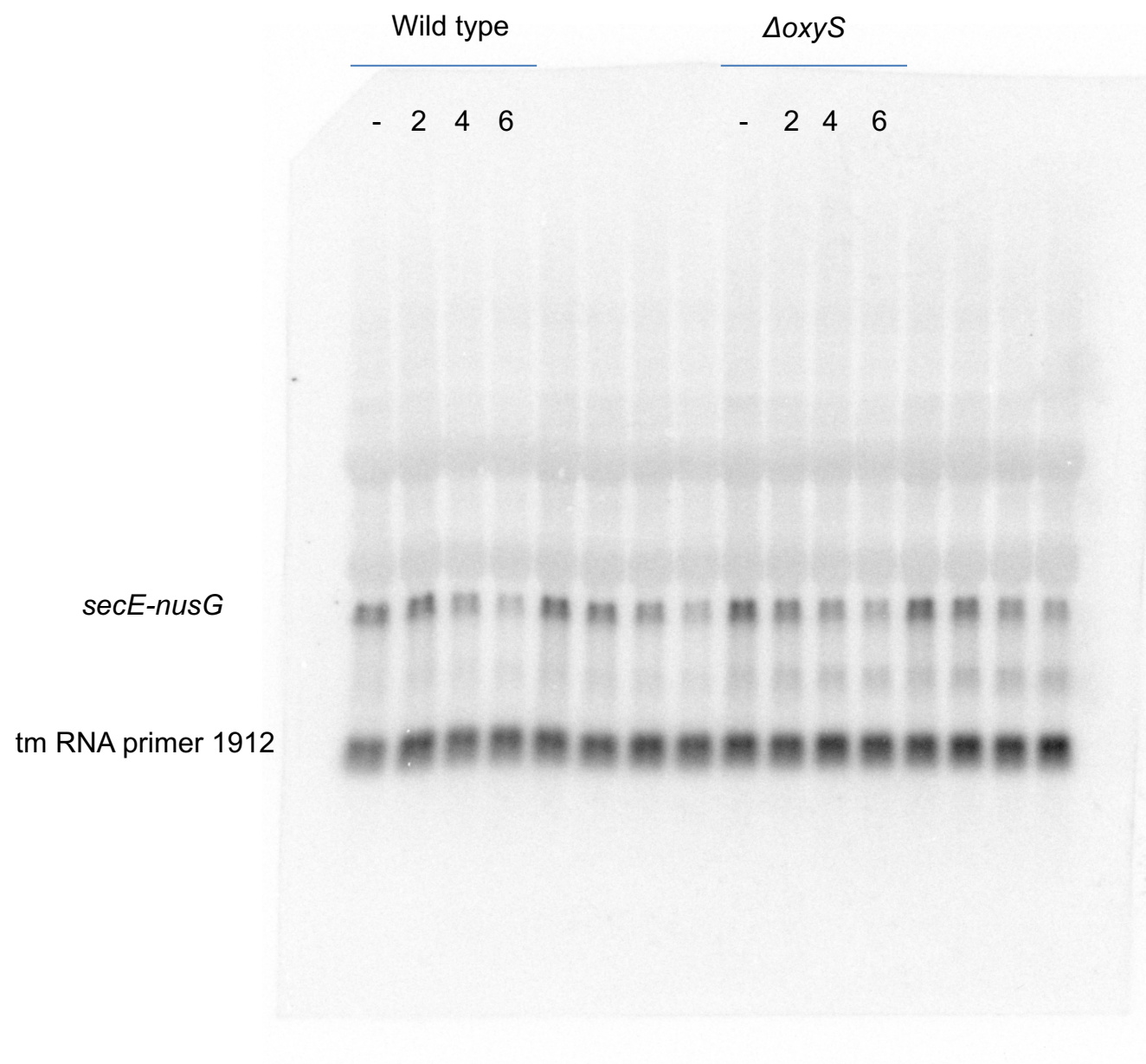

Supplement: Supplementary file 5 — Source Data for Figure 4 [file EMBJ-37-413-s003.zip › 97651_Source_Data_Fig_4B.pdf]

## Source data for Fig. 4F

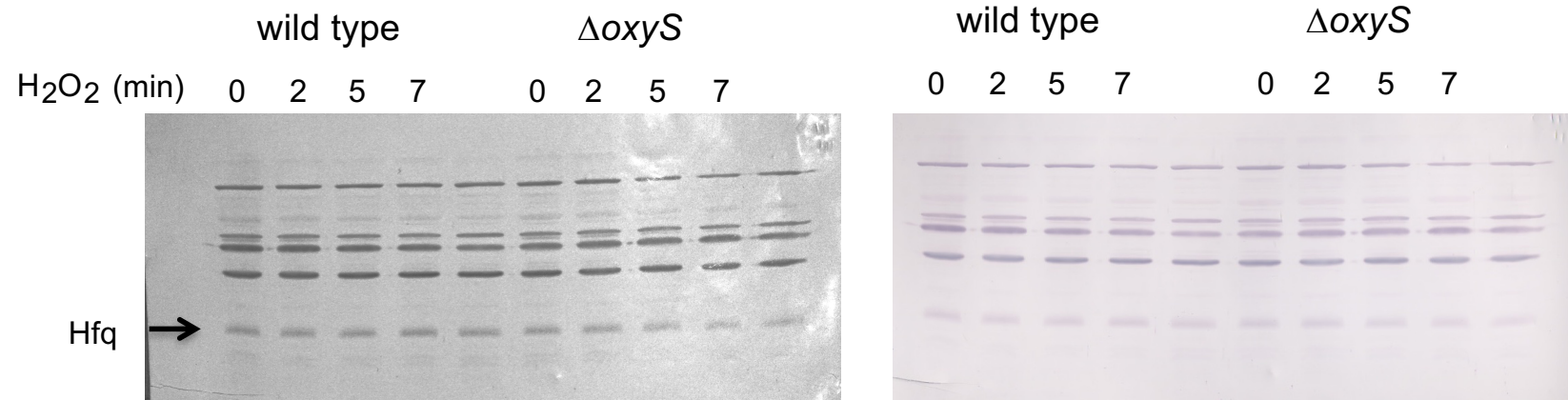

Two scans of the same blot

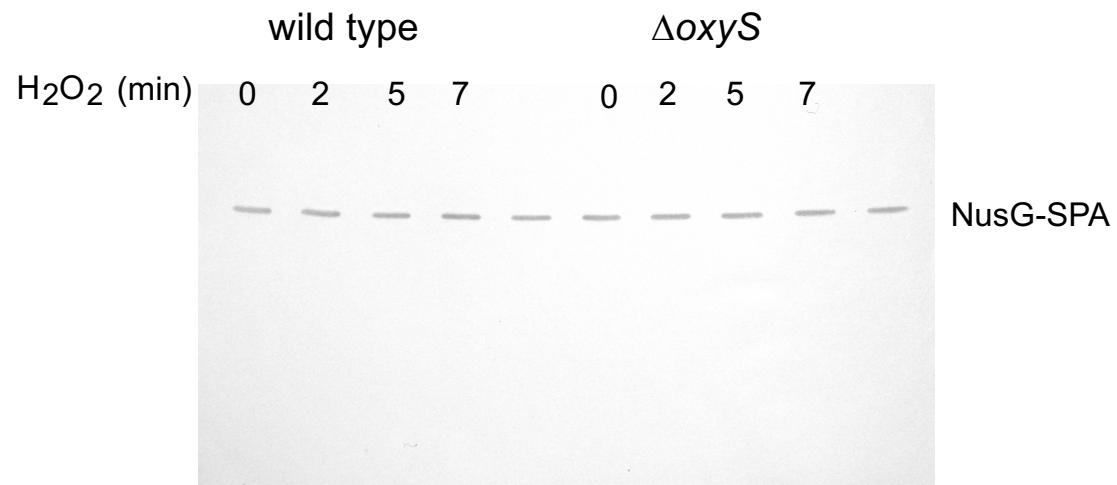

Supplement: Supplementary file 5 — Source Data for Figure 4 [file EMBJ-37-413-s003.zip › 97651_Source_Data_Fig_4F.pdf]
