# Supplementary material for: OxyS small RNA induces cell cycle arrest to allow DNA damage repair
Source: EMBO J. 2017 Dec 13;37(3):413–26. doi: 10.15252/embj.201797651 (PMC5793797; doi:10.15252/embj.201797651)
Supplement: Supplementary file 4 — Source Data for Figure 2 [file EMBJ-37-413-s002.zip › 97651_Source_Data_Fig_2B_agarose.pdf]

Source data for Fig. 2B

Northern agarose gel 1.4%
15 µg total RNA

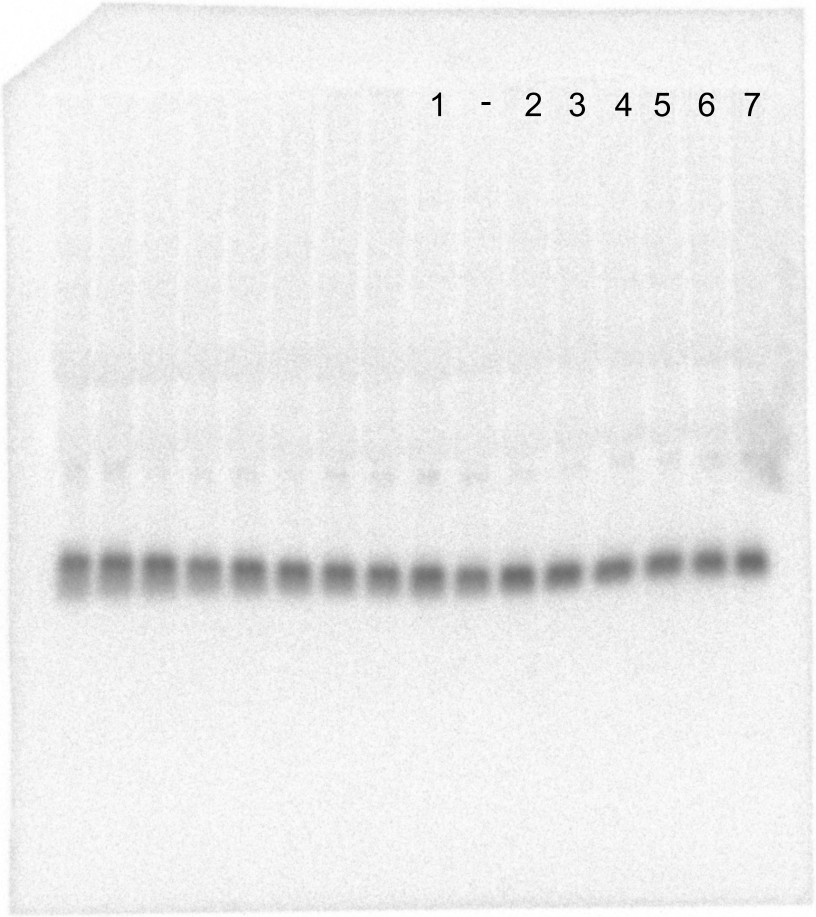

tm RNA primer 1912

1. PlacO
2. OxyS
3. OxyS A69C
4. OxyS A69C C70U
5. OxyS C56U C58U
6. OxyS C76U C77U
7. OxyS C76G C77G
