# Supplementary material for: OxyS small RNA induces cell cycle arrest to allow DNA damage repair
Source: EMBO J. 2017 Dec 13;37(3):413–26. doi: 10.15252/embj.201797651 (PMC5793797; doi:10.15252/embj.201797651)
Supplement: Supplementary file 4 — Source Data for Figure 2 [file EMBJ-37-413-s002.zip › 97651_Source_Data_Fig_2D.pdf]

Source data for Fig. 2D

6% polyacrylamide sequencing gel
Primer extension with *nusG* labeled primer (2221)

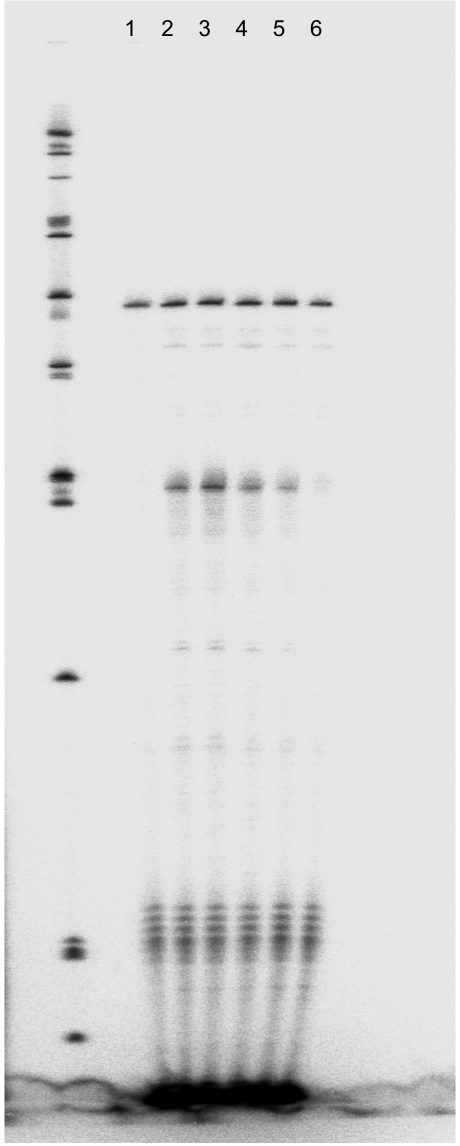

1. nusG
2. nusG+OxyS
3. nusG+OxyS A69C
4. nusG+OxyS C56U C58U
5. nusG+OxyS C76U C77U
6. nusG+OxyS C76G C77G
