# Supplementary material for: OxyS small RNA induces cell cycle arrest to allow DNA damage repair
Source: EMBO J. 2017 Dec 13;37(3):413–26. doi: 10.15252/embj.201797651 (PMC5793797; doi:10.15252/embj.201797651)

Source data for Fig. 2D

6% polyacrylamide sequencing gel
Primer extension with *nusG* labeled primer (2221)

Sequencing next to primer extension to determine the position of the termination site. Shown are the sequencing reaction and another run of the primer extension reactions

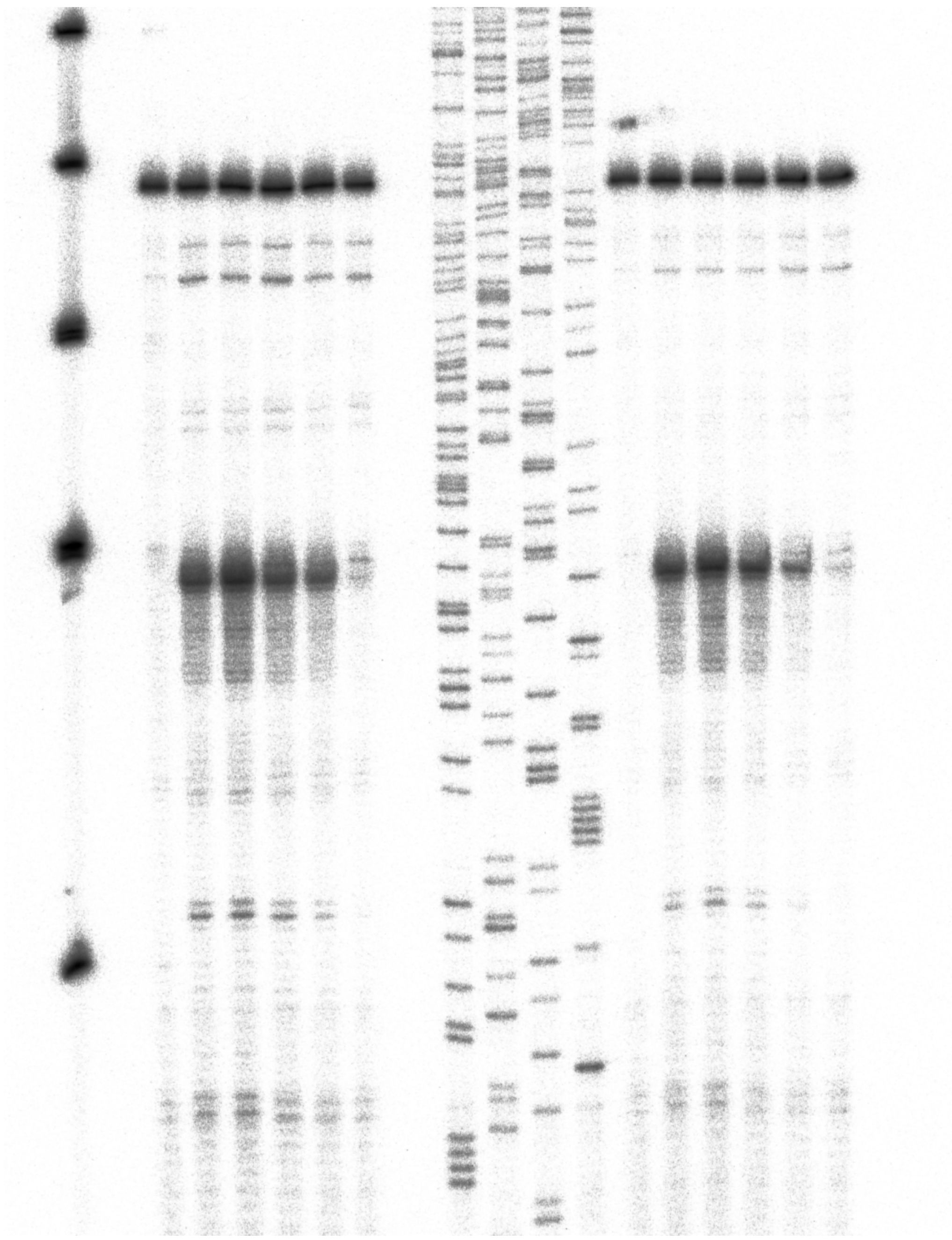

Supplement: Supplementary file 4 — Source Data for Figure 2 [file EMBJ-37-413-s002.zip › 97651_Source_Data_Fig_2D_left.pdf]
